# A Narrative Review of Neurodevelopmental, Psychiatric, and Behavioral Consequences of Preterm Birth

**DOI:** 10.7759/cureus.87423

**Published:** 2025-07-07

**Authors:** Siddharth Palanivel, Vaishnavi B Menon, Idrees Mohammed Salih, Vijayalakshmi Rajmohankumar, Khushi Sudhir Velani, Sasikala Kathiresan, Colleen Campbell, Eshita Upadhyay, Sankar Ram, Harini Pendem, Yuliya Lysak

**Affiliations:** 1 Pediatrics, GVN Riverside Multispeciality Hospital, Tiruchirappalli, IND; 2 General Medicine, Sri Ramachandra Institute of Higher Education and Research, Chennai, IND; 3 Biochemistry, Government Medical College Konni, Konni, IND; 4 General Medicine, Revathi Medical Center, Tirupur, IND; 5 Medicine and Surgery, New Vision University, Tbilisi, GEO; 6 Obstetrics and Gynecology, All India Institute of Medical Sciences (AIIMS) Madurai, Madurai, IND; 7 Pediatrics, University Hospital of the West Indies, Kingston, JAM; 8 Pediatrics, Cambridge University Hospital, Cambridge, GBR; 9 Acute Medicine, University Hospitals Birmingham, Birmingham, GBR; 10 General Medicine, Chalmeda Anand Rao Institute of Medical Sciences, Karimnagar, IND; 11 Medicine, St. George's University, St. George's, GRD

**Keywords:** developmental and behavioral pediatrician, developmental pediatrics, neurodevelopmental outcome, preterm birth, psychiatric conditions

## Abstract

Preterm infants survive into adulthood. However, this survival often comes with increased health and social challenges during adolescence and adulthood, including medical disabilities, learning difficulties, and behavioral and psychological issues. Understanding the long-term outcomes of preterm birth is crucial for developing prevention and treatment strategies, as well as setting future research priorities.

Research in this area can be complex due to the need for long follow-up periods and changes in the classification of diseases and outcomes over time, which make it challenging to establish meaningful associations. This narrative literature review aims to consolidate information about the long-term neurodevelopmental, psychiatric, and behavioral outcomes and management of individuals born preterm.

Key risk factors affecting preterm survivors include comorbid conditions and socioeconomic status. Prematurity disrupts cortical growth and myelination, making the brain vulnerable to vascular injury. These issues can lead to white matter injury, interventricular hemorrhage, and cerebellar hemorrhage, resulting in motor, language, and learning deficits. Cognitive impairments and difficulties with executive functions can adversely affect academic performance in preterm children.

Research consistently shows that early intervention and long-term follow-up can improve neurological impairment. Because the brains of preterm infants are still developing, they require continuous care to support their social and educational development. Early physiotherapy, targeted speech therapy, and individualized education programs tailored to assess school readiness can significantly enhance motor skills and mitigate the neurological impacts of prematurity.

Additionally, prenatal interventions and preventive strategies can help reduce the risk of long-term neurodevelopmental impairments. More studies with extended follow-up into adulthood are needed to better understand the social, behavioral, and psychological impacts of prematurity.

## Introduction and background

Preterm birth is defined as a birth that occurs before 37 weeks of gestation, or fewer than 259 days from the first day of the woman's last menstrual period (LMP) [1].

Each year, approximately 15 million babies are born prematurely worldwide. In 2023, the premature birth rate among babies born was 10.41%, showing little change from the previous year, 2022. Notably, the highest rates of prematurity continue to be reported among minorities: Hispanic (10.41%), Black (14.69%), Alaska Native (12.23%), and White (9.45%) infants [2].

Over the past decade, the global rate of preterm births has remained relatively stable with no noticeable change. Despite an increase in facility-based births and a strong emphasis on routine health data systems, significant gaps in accurate national routine preterm birth data still exist, especially in the high-burden regions of Southern Asia and sub-Saharan Africa [3]. Prematurity can be classified by gestational age into three groups: extreme (<28 weeks), very preterm (28-32 weeks), and moderate-to-late preterm (32-37 weeks) [3].

Research has increasingly associated adults who were born preterm with higher odds of experiencing chronic health conditions involving different organ systems, such as respiratory, cardiac, endocrine, metabolic, neurodevelopmental, and psychiatric disorders. These disorders can sometimes initially appear in adulthood or persist from childhood into adulthood. Such individuals are also at higher risk of mortality, particularly for those born at earlier gestational ages. There is a reported 30-50% increase in the mortality rate for individuals who were born preterm and are now early to mid-aged adults in comparison to individuals who were born full term [4].

Moreover, families of premature children frequently need extensive community-based care after being discharged from neonatal hospitals. Thus, further studies of prematurity are increasingly important, as medical professionals must comprehend the unique needs of parents of premature newborns [5].

An increasing body of research suggests that preterm babies are at a higher risk for a variety of neurodevelopmental abnormalities, psychiatric problems, and behavioral issues that can last into adolescence and adulthood. Despite the growing literature, these findings are often dispersed across disciplines, and a thorough synthesis is needed. Understanding the entire range of outcomes is crucial for early intervention, long-term treatment, and policy development to help this vulnerable population.

This narrative review aims to consolidate current knowledge on the neurodevelopmental, psychiatric, and behavioral consequences of preterm birth. By critically examining existing literature across clinical and developmental domains, the review aims to identify consistent patterns and emerging trends in outcomes, highlight potential mechanisms linking preterm birth to adverse developmental trajectories, and highlight research gaps that need to be addressed. Ultimately, this review offers clinicians, researchers, and policymakers a comprehensive perspective that can inform future care practices and research objectives for preterm infants.

## Review

Neurodevelopmental outcomes associated with preterm birth

Infants born prematurely face a higher risk of mortality as well as several health issues. Even though medical care has advanced, and also this vulnerable population survives at a higher rate, emerging evidence suggests that neurodevelopmental impairments (NDIs) associated with preterm birth are decreasing. These impairments include many challenges, such as cognitive deficits, neurosensory issues, delays in the development of motor coordination skills, and dysregulated behavior [6]. This section offers in-depth insights into the cognitive, sensory, and motor aspects of neurodevelopmental outcomes.

Cognition

Preterm birth is strongly associated with impairments in core cognitive domains, such as IQ, executive function, and memory, due to structural and maturational changes in specific brain regions, which can impact academic performance and self-sufficiency in preterm individuals [7].

A neuroimaging study conducted by Nosarti et al. [8] in 2014 indicates that preterm infants, especially those born very preterm (VPT) or with very low birth weight (VLBW), are at risk for cognitive deficits. These include lower full-scale IQ (FSIQ), executive function (EF), and memory performance. Significant cognitive impairments were found in VPT individuals aged 19-20 years compared to full-term controls, with lower scores in FSIQ, EF, and the non-verbal memory domain. These deficits are associated with reduced gray and white matter in specific parts of the brain, with reductions of 14%, 21%, and 17%, respectively [8]. These findings suggest a close relationship between brain volume and cognitive performance in preterm infants, underscoring the direct effects of brain changes due to preterm birth and their long-term implications for cognitive development. However, the exact mechanism by which these regions influence the different domains of cognitive function remains complex and multifaceted.

Further building on this vulnerability, the study conducted by Kim et al. highlights a significant lowering of mean FSIQs (89.1) in VLBW and extreme preterm infants (EPIs) compared to full-term infants (107.1). Additionally, EPIs with retinopathy of prematurity (ROP) who were treated using laser therapy had an 8.8-fold higher risk of low FSIQ. These findings underscore the vulnerability of preterm infants to cognitive deficits [9]. 

Focusing more specifically on memory and learning, the studies conducted by Thompson et al. [10] and Aanes et al. [11] on the influence of hippocampal growth on cognitive functions in preterm infants (gestation age (GA) <32 weeks]) highlight deficits in memory and learning during early childhood. The study by Thompson et al. [10] observed a similar growth trajectory between preterm and full-term groups in late childhood, suggesting that memory and learning deficits during early developmental stages may be mitigated through neuroplasticity, potentially reducing long-term damage. In contrast, the study by Aanes et al. [11] suggests that deficits present during early childhood will have long-term effects. This difference is attributed to the longitudinal design and more comprehensive follow-up in the study by Thompson et al. Despite the differences, both studies reinforce the notion that brain dysmaturation in the hippocampus is crucial in memory and learning difficulties in preterm infants, thereby underscoring the need for early intervention and long-term monitoring of cognitive development in these infants. 

Cognitive outcomes in late childhood and adolescence vary due to GM and WM maturation compared to hippocampal growth, with good outcomes in the latter. This suggests the need for deeper research in this arena to gain clarity on how different parts of brain development influence the various cognitive domains and therapies that might mitigate these outcomes.

Sensory and Motor Outcomes

Preterm birth is associated with impaired sensory integration and motor function, both of which are essential for achieving developmental milestones. Sensory integration helps organize sensations required for adaptive movement and motor responses. In a preterm with immature brain development, the improper processing of sensory inputs leads to dysfunctional responses, which represent sensory deficits. These deficits are attributed to a lack of inhibitory control needed to process, organize, and select sensory input. As a result, these sensory processing issues can lead to altered behavior, delayed achievement of developmental milestones, and impaired motor development [12,13]. Consequently, these sensory and motor issues extend to developmental delays across various domains, including gross motor, fine motor, language, communication, problem-solving, and social skills, which can result in poorer academic performance [14]. 

Findings from both observational and retrospective studies support the presence of these deficits in late preterm infants (LPIs), particularly in fine motor and social development. An observational cohort study by You et al. in Xi’an City, China, highlighted sensory integration dysfunction in LPIs, especially in balance and nervous system regulation, which can affect overall motor coordination. Additionally, 45.1% of LPIs showed lower scores across multiple domains, including fine motor, adaptability, language, and social development, indicating the need for early screening and intervention [15]. Similarly, a retrospective study by Martínez-Nadal et al. categorizing LPIs into complicated (cLPI) and uncomplicated (uLPI) groups based on perinatal morbidity, found that LPIs with perinatal complications (cLPI) were significantly more likely to have developmental delays (DD), especially in the communication and social domains. Moreover, cesarean section and respiratory complications were identified as significant risk factors for motor delays, highlighting the influence of perinatal complications on developmental outcomes [16].

Progression of motor skills in VLBW infants shows early delays, although outcomes may improve with intervention. Bélanger and colleagues conducted two studies on preterm infants in 2018 and 2021. The 2018 study found delays in gross motor function in VLBW infants; however, early physiotherapy contributed to improved outcomes by the end of the follow-up period. The 2021 study reported that, although most preterm infants experienced language delays, their fine motor function initially remained within the average range. Interestingly, follow-up revealed that those with early language delays later developed fine motor deficits, indicating that some developmental delays may not be evident until later, emphasizing the importance of extended follow-up [17,18].

Overall, these studies disclose a clear association between preterm birth and developmental challenges across several domains, particularly in LPIs and VLBW infants. As some delays may not be apparent in early infancy, careful follow-up is necessary to identify emerging deficits. This suggests that early screening, close monitoring, and early interventions, such as physiotherapy and targeted speech and language therapies, are essential in mitigating the long-term effects of these developmental delays in preterm infants [15-18].

Cerebral Palsy

Preterm infants are at a significantly higher risk of developing cerebral palsy (CP), a group of permanent disorders caused by non-progressive injury to the developing brain that results in activity limitations. CP is mainly characterized by motor impairment but often includes cognitive, communicative, behavioral, and musculoskeletal issues, as well as epilepsy and sensory deficits such as impaired perception, proprioception, vision, hearing, and sensory processing, all of which contribute to functional limitations in affected individuals [19,20].

Despite recent advances in medical interventions, particularly the use of antenatal magnesium sulfate, which has significantly reduced the incidence of CP, several factors continue to increase its prevalence in preterm populations [21]. For instance, Aubert et al. identified multiple risk factors such as young maternal age, male gender, and neonatal complications like bronchopulmonary dysplasia (BPD), intraventricular hemorrhage (IVH), and white matter injury, all of which greatly increase the risk of CP with adjusted relative risk ratios (aRRR) ranging from 2 to 3. These findings highlight the multifactorial nature of CP in preterm infants, with brain injuries and neonatal comorbidities making substantial contributions. Nonetheless, this study is limited to developed countries, where the incidence of CP remains relatively low compared to developing countries [22]. 

Extending these observations to developing regions, a large-scale retrospective cohort study conducted by Wang et al. in Taiwan, involving over 100,000 children, revealed a significant increase in the risk of CP in extremely low birth weight (ELBW) infants [23]. The adjusted hazard ratio (aHR) for CP in ELBW infants was 62.73 compared to full-term infants. These findings support the conclusions of the study by Aubert et al. and reflect the major challenges faced by developing nations, which are attributed to delayed diagnosis, limited healthcare infrastructure, and inconsistent follow-ups resulting in persistently high CP rates [22]. 

In addition to CP, other motor disorders and movement difficulties, such as developmental coordination disorder (DCD), are increasingly recognized in extremely preterm (EP) infants. DCD affects the ability to perform fine and gross motor tasks, such as handwriting and sports, and it often co-occurs with broader NDIs [24]. A study by Bolk et al. (2018) emphasizes that EP children have a significant risk of DCD with an adjusted odds ratio (aOR) of 7.92, underscoring the importance of assessing motor function even in the absence of CP [25].

Further supporting this, Aubert et al. identified several factors contributing to non-CP movement difficulties in EP infants, including gestational age, neonatal brain injury, postnatal interventions, and sociodemographic characteristics [24]. These findings highlight the importance of early detection, long-term follow-up, and medical intervention for motor delays that may otherwise be overlooked in children without a formal diagnosis of CP [24,25].

Indirect factors associated with NDI of preterm

While preterm birth is itself a significant risk factor for NDI, other risk factors, both environmental and biological, also negatively impact the long-term outcomes of NDI [26].

Socioeconomic status (SES), particularly maternal education, plays a crucial role. A survey by Benavente-Fernández et al. (2019) found that higher maternal education can help mitigate the adverse cognitive outcomes of brain injury, while a study by Joseph et al. revealed that lower maternal education is associated with poorer cognitive and academic outcomes by the age of 10, especially in families with no financial benefits from the government. These findings collectively underscore the strong impact of maternal education and economic support on cognitive and motor outcomes [27,28].

Alongside socioeconomic and educational backgrounds, environmental and biological factors such as inadequate antenatal care, male sex, multiple pregnancies, chorioamnionitis, bronchopulmonary dysplasia (BPD), perinatal infections, intraventricular hemorrhage, fetal growth restriction, prolonged mechanical ventilation, and inadequate postnatal growth significantly increase the risk of adverse neurodevelopmental outcomes. These factors can hinder cognitive and motor test scores [29-31].

All these studies collectively show that NDI in preterm infants is affected by both biological and environmental factors and tackling these with a combined medical and social approach is crucial to improve neurodevelopmental outcomes [27,28,29,31].

Table 1 shows the analysis of the types of behavioral outcomes studied, along with the tools used, study design, and sample size, and concludes with implications.

**Table 1:** Neurodevelopmental outcomes of preterm birth VLBW: Very low birth weight; FSIQ: full-scale IQ; EPI: extreme preterm infant; VPT: very preterm; FT: full term; ASD: autism spectrum disorder; SES: socioeconomic status

Authors	Sample Size	Study Design	Age at Assessment	Tools Used	Type of Behavioral Outcome Studied	Conclusions	Implications
Nosarti et al. [8]	302 very preterm infants	Longitudinal	Various ages (from childhood to adulthood)	Neonatal cranial ultrasound, neuropsychological assessments	Cognitive performance, brain volume	Cognitive impairments in very preterm individuals; brain volume is closely related to cognitive performance	Highlights the importance of brain development on cognitive outcomes.
Kim et al. [9]	71 EPI	Cross-sectional	Not specified	FSIQ, cognitive assessments	Cognitive development	VLBW and extreme preterm infants had lower FSIQ; laser therapy for ROP increases the risk of cognitive deficits	Identifies increased risk of cognitive deficits in preterm infants, especially with ROP treatment.
Thompson et al. [10]	125 VPT and 25 FT children	Longitudinal	Childhood	MRI, cognitive tests	Memory, learning, and hippocampal growth	Hippocampal growth influences memory and learning; neuroplasticity may reduce deficits	Potential for neuroplasticity to reduce cognitive deficits in later childhood.
You et al. [15]	215 LPI infants	Cross-sectional	1-3 years	Sensory and developmental assessments	Sensory integration, ASD, and developmental scores	LPI infants had sensory integration dysfunction and developmental delays; 8.8% positive for ASD	Highlights the need for early intervention for sensory and developmental issues in LPI infants.
Bélanger, [17]; Bélanger et al., [18]	Not specified	Longitudinal	Infancy to toddler years	Motor and language assessments, physiotherapy	Motor development and language delay	Gross motor and language delays in VLBW infants; early physiotherapy improved outcomes	Emphasizes early physiotherapy to address motor delays in preterm infants.
Martínez-Nadal et al. [16]	163 LPI - 47 cLPI and 116 uLPI - and 158 term-born infants	Retrospective	Infancy	Developmental assessments	Communication and social development	LPI infants are at greater risk for communication and social delays, especially with respiratory complications	Suggests targeted interventions for preterm infants with respiratory complications to reduce developmental risks.
Aubert et al. [22]	366 children without movement difficulties (MD), 100 children with cerebral palsy (CP), and 224 children with non-CP MD.	Retrospective	Various ages	Medical data and CP assessments	Cerebral palsy (CP)	Identified risk factors for CP, including maternal age, male sex, BPD, and brain injuries	It supports the need to identify high-risk infants to prevent or mitigate CP.
Wang et al. [23]	1,000,000 children	Retrospective	Various ages	CP assessments and medical data	CP	ELBW infants had the highest CP incidence	Calls for improved monitoring and interventions for ELBW infants to prevent CP.
Bolk et al. [25]	229 preterm children and 344 term	Cross-sectional	School-age	Cognitive, language, and behavioral assessments	Developmental coordination disorder (DCD), cognitive, and language issues	Extremely preterm children at higher risk for DCD; co-occurring issues with cognition and language	Emphasizes early identification of DCD in preterm children for intervention.
Aubert et al. [24]	772 children born extremely preterm	Retrospective	Various ages	Movement difficulty assessments and medical data	Motor difficulties and movement disorders	Extremely preterm infants had an increased risk of movement difficulties linked to brain injury, GA, and corticosteroid treatment	Highlights the need for monitoring and interventions to address movement difficulties in preterm infants.
Benavente-Fernández et al. [30]	226 preterm neonates born at 24–32 weeks	Retrospective	Various ages	Cognitive assessments and maternal education data	Cognitive development	Higher maternal education reduced the negative effects of brain injury on cognitive outcomes	Supports the importance of maternal education in improving cognitive outcomes in preterm infants.
Joseph et al. [27]	440 extremely preterm infants	Longitudinal	Age 10	Cognitive assessments	Cognitive development and SES effects	Lower maternal education and SES negatively impacted cognitive outcomes by age 10	Highlights the need to address SES disparities for better cognitive outcomes in preterm infants.
Li et al. [31]	1,000 extremely preterm infants	Retrospective	Various ages	Perinatal data and neurodevelopmental assessments	Neurodevelopmental outcomes	Perinatal factors like chorioamnionitis, BPD, and mechanical ventilation increase the risk of adverse outcomes	Indicates the importance of addressing perinatal risk factors to prevent adverse neurodevelopmental outcomes.

Psychiatric outcomes associated with preterm birth

Children born preterm face a higher risk of developing mental health conditions and educational challenges throughout childhood. Preterm birth is significantly linked to an increased risk of autism spectrum disorder (ASD), attention-deficit hyperactivity disorder (ADHD), cerebral palsy (CP), nonaffective psychosis, depressive disorders, and bipolar affective disorder [32]. Additionally, the literature also showed an increased risk of developmental delays and learning difficulties. Studies also showed that very preterm birth increases the risk of developing these conditions [33].

Attention-Deficit Hyperactivity Disorder

In very preterm children (gestational age< 33 weeks), various ADHD symptoms have been identified, particularly during preschool years. Notably, these symptoms can differ from typical presentations observed in term-born children. A significant portion of very preterm children, approximately 32.7%, met the criteria for subthreshold ADHD inattentive type. In contrast, about 33.6% exhibited symptoms characteristic of combined type ADHD, which includes both inattentive and hyperactive/impulsive symptoms, which were consistent with other studies [34]. ADHD in these children varied from typical presentations; for example, inattention was identified as a core deficit, and further studies failed to reveal a subgroup with predominantly hyperactive/impulsive symptoms. Also, higher ADHD symptom scores correlated with greater executive dysfunction, including issues with inhibitory self-control and flexibility [35]. *Autism Spectrum Disorder*

Preterm birth has also been linked to an increased incidence of ASD in later stages of life [34,36-38]. A cohort study conducted by Hack et al. in which ASD incidence in 219 extremely low-birth babies was compared to 176 term-born babies using DSM-IV criteria [34]. The study concluded preterm babies had an odds ratio of 3 compared to term babies. The study also stated no association between preterm and risk of Asperger’s syndrome, which can be attributed to diagnostic criteria unlikely to be fulfilled due to the risk of high levels of neurodevelopmental delay in very preterm babies. Existing literature shows an evident association between preterm birth and ASD, irrespective of sample size, geography, sex, and other risk factors [39]. 

Mental Health Disorders

Studies have shown a significant association between preterm birth and decreasing gestational age with the incidence of conditions like nonaffective psychosis, depressive disorders, bipolar diseases, eating disorders, and drug dependency [32,40]. Several studies on ASD in preterm babies have also shown a positive correlation between mental health disorders and decreasing gestational age [34,39,41]. Studies also demonstrated an increased incidence of psychiatric symptoms and behavioral problems in preterm children diagnosed with ASD [42]. Psychiatric disorders were also found to be associated with poor cognition and low IQ scores across various studies [34]. The underlying pathophysiology associated with preterm, especially extreme preterm, such as focal brain injury and altered brain development, can increase the incidence of both psychiatric disorders and cognitive impairments. However, it should be noted that most are found in association with other morbidities, particularly cognitive impairment [40].

Factors affecting psychiatric outcomes of preterm birth

The long-term psychiatric outcomes in pre-term babies are influenced by a multitude of factors, including gestational age at the time of birth, complications of prematurity, mental health of the mother, SES, and parental education. Many factors contributing to preterm birth can also affect the long-term outcomes of these conditions, making it challenging to establish a causality relationship. The processes leading to preterm birth, as well as the clinical management needed to sustain life in pre-term babies, especially extreme pre-term, such as Invasive Mechanical Ventilation, can have adverse outcomes later in life [36,40]. 

A study conducted in Sweden, which included a total of 4061795 participants, was able to illustrate factors affecting ASD in pre-term babies [36]. Factors negatively affecting the long-term outcomes included gestational age of birth, birth weight, maternal comorbidities, and maternal lifestyle, including smoking habits and substance abuse. Extreme preterm babies, small for gestational age, maternal diabetes, and hypertension were found to have significant associations with poor outcomes in children. Meanwhile, parents' socio-economic and educational status, access to healthcare, and good support systems were found to have positive outcomes, including early diagnosis and early interventions in these children. It was also noted that stressful living environments, including exposure to violence or instability, can adversely affect child development and health outcomes. This study provided keen insights into the outcome of preterm babies. A large time frame from 1973 to 2013 and a large sample size make this study highly reliable, but a lack of generalizability and detailed clinical records limit the applicability of the study to a diverse population. The study focused more on the prevalence of ASD in children up to age 2. Hence, further studies for long-term follow-up are required, which highlight areas for further research and caution in interpreting the findings in broader contexts. It is also to be noted that the factors associated with increased risk of preterm and prematurity were also associated with increased risk of ASD [43].

Additional factors associated with increased psychiatric risk include advanced parental age, genetic predispositions, cesarean delivery, male gender, and the presence of other developmental disorders. The necessity of invasive mechanical ventilation in preterm infants is correlated with higher risks for intellectual disabilities and ASD. For instance, children born at 27 weeks gestation are approximately eight times more likely to experience intellectual disabilities than term-born peers. Despite the small sample size and potential biases in follow-up participation, these findings underscore the need for further investigation into causative mechanisms [44].

Children who develop social skills and communication skills earlier tend to have better long-term outcomes. Interventions focusing on social communication can significantly improve prognosis. Programs that enhance peer interactions and social understanding, such as comprehensive educational support, can improve social integration in later life. In preterm children diagnosed with ADHD, early intervention correlates with positive neurodevelopmental outcomes. A study of 51 infants demonstrated significant declines in cognitive and motor scores from 6 to 24 months, highlighting the importance of routine surveillance and public health initiatives to support school readiness [45,46].

Existing literature on ADHD was synonymous with that of ASD; factors affecting positively and negatively remained relatively the same. Extreme preterm, low birth weight babies, days of mechanical ventilation, and days of parenteral nutrition were directly found to have a detrimental effect on long-term outcomes. There was a negative correlation between inattentive symptoms and IQ levels, indicating that children with more inattention tended to have lower cognitive scores. However, hyperactive symptoms were associated with lower SES, suggesting that environmental factors also play a role. Perinatal medical complications, abnormal MRI findings, and the presence of neurodevelopmental disorders also contributed to a negative outcome at a later stage. SES, parental harmony, support systems, and maternal education contributed positively to babies with ADHD, analogous to the literature on ASD [35,47]. Mothers of preterm infants often experience heightened psychosocial stress, which can adversely affect their children’s cognitive development. Maternal support and intervention programs are paramount to mitigating cognitive risks linked to depression and social disadvantage, emphasizing the importance of comprehensive family-centered care [48].

**Figure 1:**
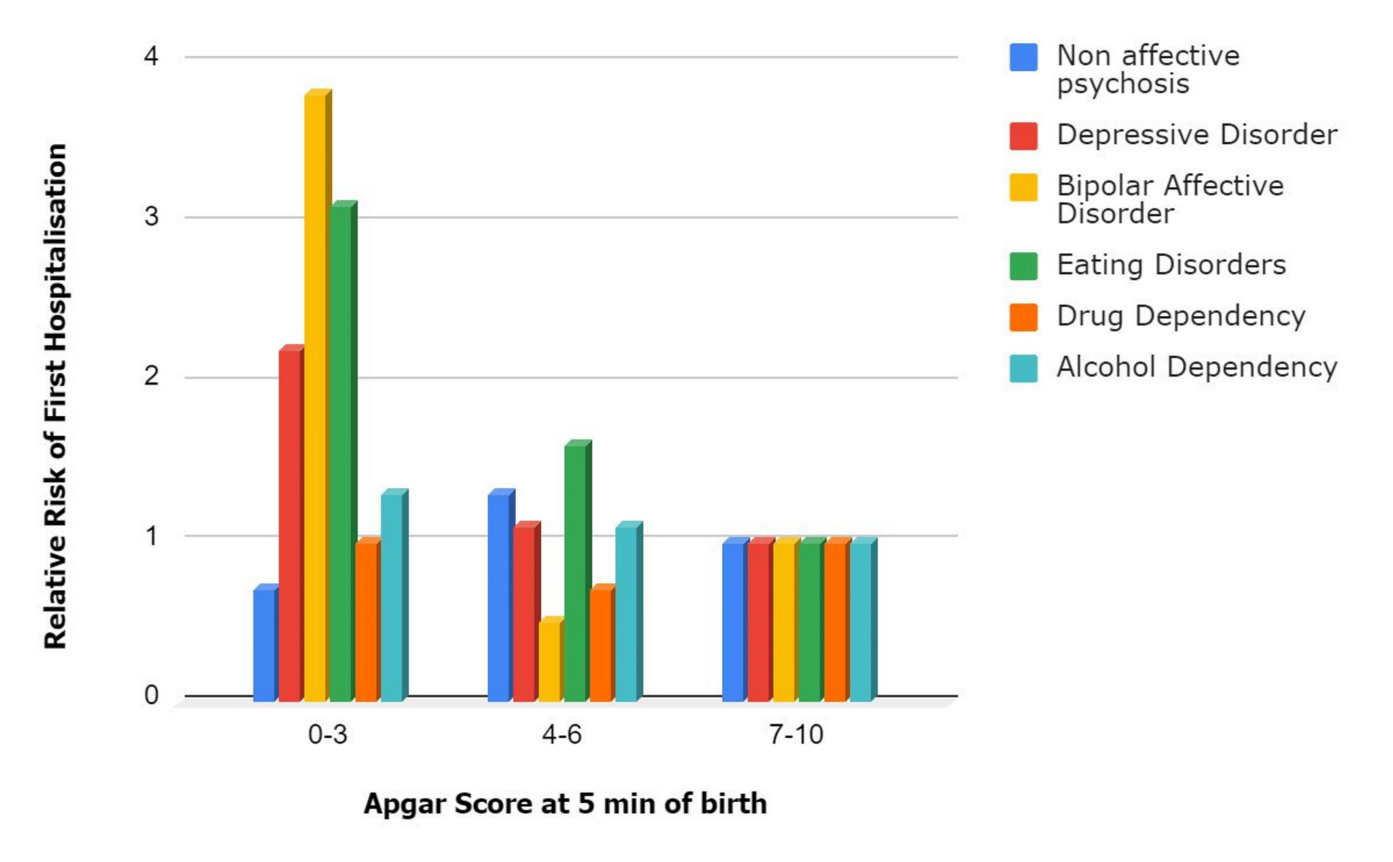
Adjusted relative risks for incidence of first hospitalization with a selected psychiatric diagnosis after an individual’s 16th birthday about pregnancy outcomes. Created using Microsoft Word with data from reference [32] (Copyrights (October 25, 2011). Archives of General Psychiatry)

A large cohort study by Nosarti et al. [32], examining 1,301,522 individuals using Swedish national health registers, demonstrated that preterm birth is significantly associated with increased psychiatric hospitalizations in adulthood, including nonaffective psychosis, depressive disorder, and bipolar disorder, and the risk was increased in extremely preterm babies. Individuals born at 32 to 36 weeks of gestation are 1.6 times more likely to have nonaffective psychosis, 1.3 times more likely to have depressive disorder, and 2.7 times more likely to have bipolar affective disorder compared to those born at term (37-41 weeks). The rates of hospitalization for depressive disorders were also affected by the condition of the newborn, reflected by the APGAR score at five minutes of birth. Other factors affecting the long-term outcomes were synonymous with existing literature, such as good socioeconomic status, maternal health, and education, showing positive outcomes in the future. Although the study had a large sample size and utilized data from a large period, certain limitations were noticed. The major limitation noticed was a possible selection bias since the study focused on psychiatric diagnoses related to hospitalization, which meant only more severe cases of psychiatric disorders were included. An underestimation of the prevalence of psychiatric disorders in the general population can be argued, as many individuals with less severe conditions may not require hospitalization. Figure 1 shows a graphic representation of the relative risk of first hospitalization of various psychiatric diagnoses. 

Several studies find a positive correlation between preterm birth and the occurrence of psychiatric disorders later in life; however, most of the studies were limited by small sample sizes and a lack of specific guidelines on diagnosing specific conditions [49]. More literature was found on the correlation between maternal mental conditions and the occurrence of preterm delivery in these individuals. Further studies are required to confidently analyze the correlation between preterm delivery and outcomes of specific mental disorders later in life [50-52].

In conclusion, factors such as extreme prematurity, low gestational age, maternal substance use, prolonged neonatal care interventions, and low Apgar scores have been found to negatively impact long-term psychiatric outcomes in preterm children. Conversely, positive factors, including parental education, socioeconomic stability, access to healthcare, supportive family environments, and specialized schools, significantly improve outcomes [45,46].

Behavioral outcomes associated with preterm birth 

Behavioral problems in children consist of a multitude of difficulties that include internalizing and externalizing behavioral profiles. Anxiety, withdrawal, depression, and dysphoria are some characteristic features of internalizing behavior, while externalizing behaviors are expressed as defiance, hyperactivity, impulsivity, aggressiveness, and disruptiveness [52,53].

A national retrospective cohort study in China showed that children between three and five years of age with a longer gestational age until 40 weeks of gestation had a beneficial outcome of neurodevelopment in early childhood, while pre-term and post-term births had a higher risk of neurodevelopmental delay [54]. This relationship suggests that the timing of elective preterm delivery should be carefully considered. Although neonatal care has improved with increased survival of preterm births, the long-term sequelae need further assessment [55].

"Preterm behavioral phenotype" is a term described by Johnson and Marlow that characterizes symptoms like inattention/hyperactivity and social and emotional difficulties, seen more often in lower gestational ages in their study of preterm births and childhood psychiatric disorders. They also stated that these children were more likely to exhibit signs of internalizing behavior rather than externalizing problems [41].

Screening tools such as the Child Behavioral Checklist (CBCL) and the Strengths and Difficulties Questionnaire (SDQ) are used at a large scale to assess behavioral morbidity as they are time- and cost-effective assessments [56]. 

During Childhood

Published findings have shown that very preterm-born children show higher scores on CBCL anxiety/depression and SDQ emotional difficulties scales, indicating significant emotional difficulties relative to their peers. These issues with behavioral self-regulation have been shown to have an impact on preterm toddlers, affecting their interactions with age-matched peers and social competence appropriate for their age. Higher scores on peer problems have been consistently noted through these screening questionnaires and imply impaired social skills and social withdrawals in very preterm children during preschool and school age. Neurodevelopmental delays in preterm children are a possible way through which behavioral problems may arise, specifically cognitive and learning difficulties affecting school performance and, in turn, contributing to them feeling victimized or socially excluded [56].

Girls born late preterm and early term are at a heightened risk of emotional problems at 36 months of age, indicating that gender should be considered an aspect of differentiation when assessing those born in these gestational ages [57].

Williamson et al. showed that VLBW preterm children have difficulty interpreting nonverbal cues such as facial, body movements, and situational cues, as well as poor performance on the CASP (Child and Adolescent Perception Measure). The study did not address whether these issues lead to future behavioral problems; however, it highlighted the importance of conducting comprehensive assessments in survivors of very preterm birth, particularly those who face social difficulties despite functioning within the normal intellectual range [58]. Similarly, an investigation in Sweden assessing children aged 10-15 years using parent-teacher reports showed that children born early preterm had higher ratings of internalizing behavior and social issues than their full-term peers [59].

A meta-analysis by Aarnoudse-Moens et al. showed a comparable neurobehavioral outcome in very preterm and/or VLBW children, with teachers noting more problems with attention and internalizing behavior. In addition, there was also no significant increase in the rating of externalizing behavior among very preterm and/or VLBW children [60]. Raised anxiety/depression scores have been consistently reported for children aged 8-11 years who were born extremely preterm using both parent and teacher reports [61].

Korzeniewski et al. used the Social Responsiveness Scale (SRS) to assess the "typical" social characteristics of the “preterm behavioral phenotype” and their severity in ten-year-olds. This scale is comprised of questions about five main domains: social awareness, social cognition, social communication, social motivation, and autistic mannerisms. The study's results indicated that the prevalence of social impairment defined by the SRS was four times higher than expected among 10-year-olds born before the 28th week of gestation compared to the general population. This finding holds even after excluding children who met the diagnostic criteria for ASD at age 10, with rates of 16% for those with an IQ of 85 or higher and 27% for those with an IQ below 85, compared to just 4% in the general population [62]. 

Some studies even assessed these changes and outcomes on MRI imaging, further proving that pre-term births showed a significant difference compared to their full-term peers. When comparing school-age children born late preterm and full term, with no differences in total brain volume, a study by Rogers et al. showed that late preterm children had a smaller percentage of their brain volumes as grey matter, specifically in the right temporal and parietal lobes. This, with the association in the cohort study that late preterm children had more anxiety symptoms than the full-term, suggested that decreased grey matter volume could be a reason for increased anxiety risk in that group of children [63].

Another study used diffusion tensor imaging (DTI), a magnetic resonance imaging technique that provides quantitative characterization of white matter tracts in the brain, to compare the degree of association between parent-rated scores of attention, internalizing, and externalizing behavior to white matter fractional anisotropy in children born preterm. The analysis showed that unfavorable attention and internalizing behavior scores in preterm children were consistently associated with lower fractional anisotropy. Significant associations with externalizing behavior were also seen in some areas of interest. Full-term children, on the other hand, showed no significant associations between fractional anisotropy and behavior. In addition, the associated brain regions of inattention and anxiety were highly overlapping, suggesting a common underlying neurobiology in preterm children. This has clinical implications that premature children with behavioral difficulties should be screened and assessed early [64].

Table 2 shows the behavior outcomes of preterm birth, including the type of behavioral outcomes studied, the tools used, the age of assessment, the study design, and the sample size. It also includes a conclusion and implications. 

**Table 2:** Behavioral outcomes of preterm birth. VLBW: Very low birth weight

Authors	Sample Size	Study Design	Age at Assessment	Tools Used	Type of Behavioral Outcome Studied	Conclusions	Implications
Stene-Larsen et al. [57]	43,297 preterm and early-term children and their mothers	Longitudinal cohort	36 months	CBCL and ITSEA questionnaires at 17th, and 30thweeks of pregnancy and at children age of 6, 18, and 36 months	Emotional and behavioral outcomes	Girls born late preterm and early term show an increased risk of emotional problems at 36 months	Gender is to be considered as a part of the screening of preterm births and long-term monitoring to track the progress of such emotional and behavioral problems
Williamson and Jakobson [58]	34 preterm-born children with age-matched full-term controls	Cross-sectional	8-11 years	Child and Adolescent Social Perception Measure (CASP)	Social skills, nonverbal communication	Preterm children have impaired social perception and struggle with nonverbal cues with poor performance on the CASP	Screening social skills in preterm children early can help identify difficulties and allow for targeted intervention
Aarnoudse-Moens et al. [60]	4,125 very preterm/VLBW children and 3,197 term-born controls	Meta-analysis of observational studies	5.9-17.3 years	Meta-analysis from original articles	Attention, internalizing behavior	Teachers noted more attention and internalizing problems in preterm/VLBW children vs. term peers	School-based specialized education programs that help aid attention can be inculcated early in their learning
Rogers et al. [63]	108 late preterm (34-36 weeks) and full-term children	Longitudinal	School-age, with 3-6 annual assessments	Preschool-Age Psychiatric Assessment (PAPA), Childhood and Adolescent Psychiatric Assessment (CAPA), MRI Brain	Anxiety, brain volume	Late preterm children had smaller brain volumes as gray matter and more anxiety symptoms than full-term, suggesting that decreased grey matter volume could be a reason for increased anxiety risk in that group of children	Develop targeted anxiety interventions for late preterm children, provide education to parents to understand the potential challenges, and implement the appropriate help required
Loe et al. [64]	25 born <36 weeks and 20 full-term children	Cross-sectional	9-16 years	MRI Brain with Tract-Based Spatial Statistics, DTI	Attention, internalizing behavior	Poor attention/internalizing behavior linked to lower fractional anisotropy	Understanding the implications of specific white matter changes in preterm children and being able to provide early intervention
Korzeniewski et al. [62]	1,506 children <28 weeks enrolled, 889 assessed	Multi-center, observational	10 years	Social Responsiveness Scale, Social Communication Questionnaire (SCQ)	Social responsiveness, ASD-like traits	High prevalence of social impairment in extremely preterm children	Track milestones and implement screening for social impairments in preterm care monitoring

During Adolescence

Most case-control and birth cohort studies studying teenagers have described preterm adolescents as being emotionally vulnerable compared to controls. However, various other confounding factors at play must be accounted for when it comes to assessing anxiety and depression in adolescents. The what, when, and who of these disorders, as well as how they arise in term-born peers, are important aspects to consider for a comprehensive understanding of their development and differences compared to preterm children. Anxiety shows a more typical onset in childhood, while depression emerges mostly in adolescence. Women are more likely to suffer from depression than men, and anxiety, often being a precursor to depression, makes it difficult to investigate separately. Another issue that complicates the results is how the interpretation of teachers and parents of their adolescents may not always be accurate, with parents of VLBW adolescents reporting more emotional difficulties in their children compared to parents of term-born teens, whereas the VLBW adolescents in question declared less emotional problems than controls [56].

Factors affecting behavioral outcomes of preterm birth

Factors that Positively Impact Behavioral Outcomes in Preterm Births

Parents play an important role and are currently at the center of care. Positive parent interaction and protection against early life stress seem to reduce the negative effects of neonatal pain on stress-sensitive behaviors in this vulnerable population [57,65]. Intervention programs that focus on decreasing stress in the parents of preterm infants also reduce the risk of maternal postpartum depression [66].

Early assessments and evaluations to identify learning difficulties or behavioral changes need to be done among pre-term born children in schools, and the educational sector should ensure and encourage focused programs and support started early after these evaluations [55]. School-based executive function group training has been shown to be more effective than individual training, as peer motivation and interaction with other children allow them to develop with significant support [67].

Family-based interventions are essential in scenarios where poor family-child relationships and maternal nurturance fail to enable a safe environment for the child [68].

Finally, assessing the causes of preterm births and ensuring safe pregnancies that may reach term should be the number one priority to allow for safe mothers and children with minimal effort.

Factors That Negatively Impact Behavioral Outcomes in Preterm Births

Medical complications and pain: Very preterm birth exposes infants to prolonged and repeated pain-related procedures as part of their care in the neonatal intensive care unit, as well as a risk for medical complications. A study was done to show the impact of repeated procedural pain-related stress in infants born very preterm, where neonatal pain was defined as the number of skin-breaking procedures. Neonatal pain assessments include a variety of behavioral and physiological responses such as facial actions, body movements, crying, heart rate, respiratory rate, blood pressure, and oxygen saturation to quantify pain in nonverbal patients. However, these may not necessarily indicate pain but rather agitation or distress. It becomes difficult for physicians to differentiate and manage the pain appropriately, and often, invasive procedures are still performed without support. These repeated painful procedures have been found to be associated with altered stress hormone (cortisol) regulation and lower motor and cognitive functions at 8 and 18 months corrected. Very preterm children displayed greater internalizing behaviors compared to full-term control children [56,65,69].

Role of Parents

The parent-child relationship plays a significant role in cultivating positive social behavior and self-regulatory experiences. The poor mental health of the parents, like maternal depression, has shown reduced positive affect in children as young as six months of age, showing a further predisposition in preterm children as compared to term-born [56,70]. One study showed that parents of children born very preterm had been described as having more psychological distress and, therefore, may be risk factors for the social, behavioral, and functional development of five-year-old preterm children. The clinical implication of the study is that while assessing the growth and development of preterm children, it is also essential to address the mental well-being of the parents [71].

**Figure 2:**
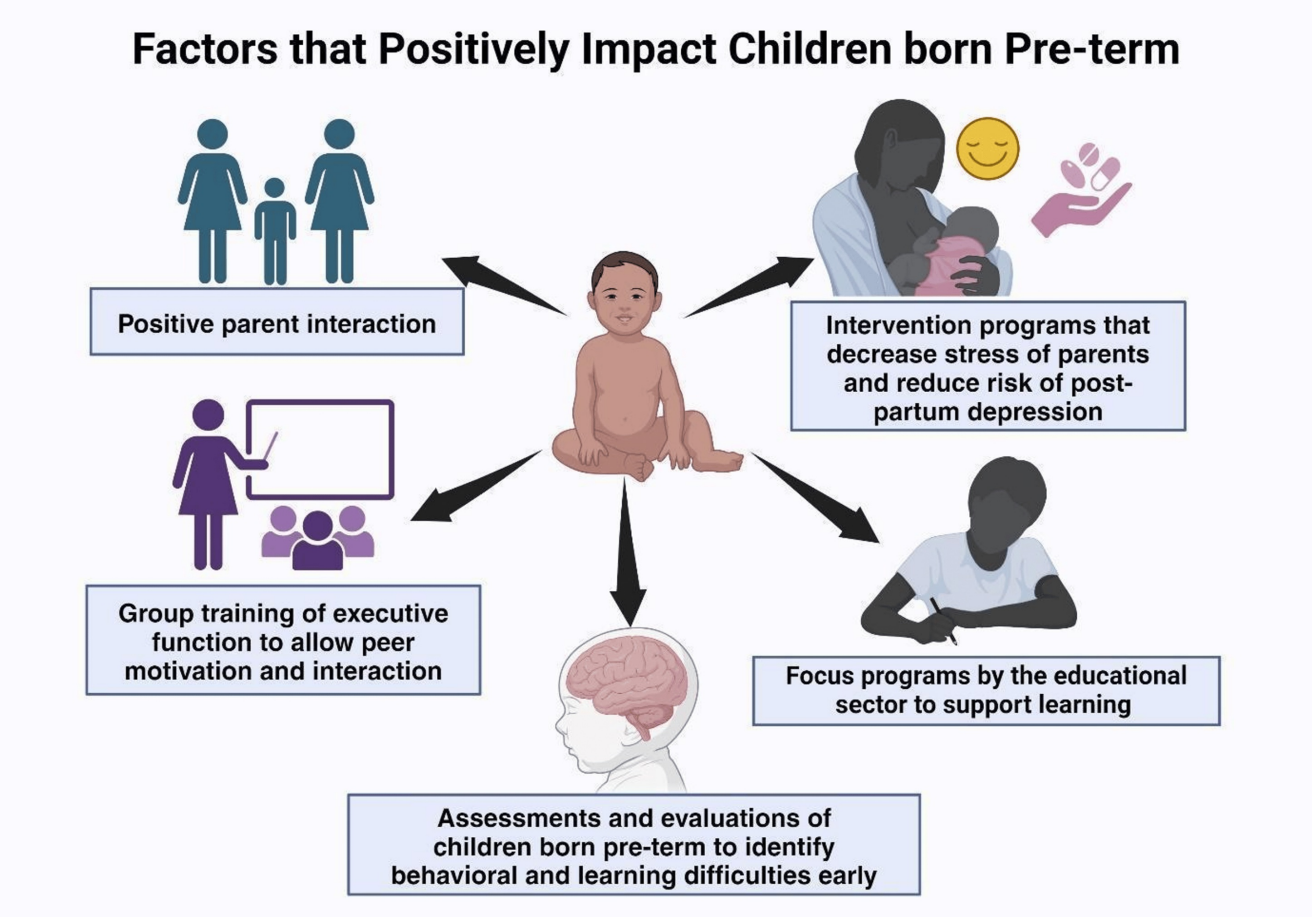
Factors that positively impact behavioral outcomes in preterm birth. Created using BioRender.com

Other than the preterm birth itself, which may contribute to problems, factors such as younger mothers, primary caregivers with lower education levels, and a higher social risk score were more commonly seen in preterm births than in term births. These variables have demonstrated early internalizing difficulties, which strongly contribute to later emotional symptom problems, as most of them occur within the first year of life [62,72]. Pascoe et al. illustrated the pivotal influence of a mother’s nurture and a good family relationship, as family avoidance, exposure to family conflict, and hostile and non-nurturing home environments are major contributors to the increased likelihood of externalizing behavioral problems in a preterm sample [68]. Figure 2 represents the factors that positively impact behavioral outcomes in preterm birth.

Management

Early Assessment and Identification

Neuroimaging: Initial imaging technologies such as cranial ultrasound, MRI, and CT scans can detect brain anomalies that increase the risk of developing neurodevelopmental disorders. In contrast, MRI can detect white matter damage (which applies to motor and cognitive deﬁcits) [73]. 

Neonatal Intensive Care Unit (NICU) screening: Despite general international agreement on the importance of screening, economic pressures and underdeveloped monitoring are obstacles to its wide use.

Screening: Assessments for conditions include IVH, periventricular leukomalacia (PVL), and retinopathy of prematurity [74].

Developmental assessments: Regular developmental assessments with standardized tools (Bayley Scales of Infant and Toddler Development) are critical to detecting delays in motor, cognitive, language, and social-emotional growth. These should start in the first year of life and be repeated periodically [75].

Medical Management

Neuroprotective strategies: In the streptococcus NICU, neuroprotective practices include minimizing stress and optimizing nutrition (using breast milk), which also require neuroprotection agents (like magnesium sulfate for very preterm infants) [73].

Nutritional support: Food and nutrition are important as they provide the energy to grow and promote brain development. Like infants, children born preterm need sufficient nutrition to help with growth. Nutritionists will plan a balanced meal routine and recommend how much each family member should eat per meal [73,75]. Prevention of preterm birth by optimizing antenatal care, managing maternal health conditions, and prophylactic treatment with interventions such as antenatal corticosteroids can decrease the risk of the neurodevelopmental disability chain [73].

Follow-up and Post-Treatment Monitoring

Ongoing developmental surveillance: Even though preterm children exit the NICU, they continue to require longitudinal developmental surveillance. This may involve regular monitoring by pediatricians and neurodevelopment specialists, as well as potentially psychologists who evaluate cognitive, motor/language, and social-emotional development [75].

School-ready assessments: Preterm children ought to be assessed before beginning school because of their school readiness. These assessments may include cognitive abilities, language readiness for school, and social interactions, which will allow schools to determine if they need accommodations or individualized education plans (IEPs) [76].

Comprehensive Rehabilitation and Therapy Services

Physical therapy (PT): This is crucial for treating motor delays and reducing the likelihood of disorders such as CP. PT is commonly a part of early intervention programs to build muscle strength, coordination, and mobility. Neurodevelopmental treatment is frequently utilized to improve motor control [75]. 

Occupational therapy (OT): OT works on fine motor, sensory processing, and activities of daily living. For preterm infants, this therapy is crucial in enabling the child to grasp and suck properly, thereby aiding in feeding as well as self-care activities in the future [75].

Speech and language therapy: Early speech and language therapy supports preterm babies who are experiencing communication delays. These interventions can target both receptive (understanding) and expressive language skills from an early age. The activities involved focus on enhancing oral-motor skills, which are important for both speech and feeding [77].

Specialized Therapies

Behavioral/cognitive therapies: As preterm children get older, they can benefit from cognitive-behavioral therapy-based interventions for ADHD or anxiety as an adjuvant treatment modality. Such therapies can help in addressing the problems linked to attention, self-regulation, and coping [78].

Social skills training: This treatment method is useful for children who shy away from social signals or have problems making friends with other kids. It is often provided in a group setting and focuses on improving communication, empathy, or problem-solving skills [79].

Educational Support Strategies

IEPs: Children with LD or cognitive impairments in preterm birth may also require IEPs when they first attend school. The IEPs are a set of activities made specifically for children that focus on the child's strengths. Every IEP uses the child’s specific needs to aid them in academic success [76].

Early childhood education programs: Children born preterm need a good environment that supports the necessary cognitive and social growth. Some countries have developed special programs for children at risk, such as the U.S.’s Head Start, a program developed to meet the needs of children born preterm [76].

Support and Counselling for Parents

Parental education: It is critical to educate parents about the potential neurodevelopmental sequelae their premature infant may encounter. This also includes coaching on how to engage positively with their child (e.g., play and communication, nurturing interactions) to promote healthy development. Several family-centered care programs have been introduced, offering educational support to boost their confidence [79].

Counselling services: For the high stress and anxiety experienced by parents of preterm infants, both parents need support, especially emotionally and in the child's development, and therapy services are highly beneficial for developing effective coping strategies. One strategy to enhance parental involvement and bonding is the family-centered care approach in the NICU, which encourages active presence in caring for the baby, which as a whole improves the mental health and helps the parents handle challenging situations [79].

Long-Term Management and Cure

Integrated care teams: Neurodevelopmental management of infants born preterm requires a multidisciplinary approach. Care is coordinated using a multi-specialist approach, working collaboratively. Children have access to an interdisciplinary team, including pediatricians, neurologists, PT/OT/Speech therapists, educators, and mental health professionals, hence providing continuity of care [75,77,79].

## Conclusions

Preterm birth is one of the major causes of morbidity and mortality in infants. Brain injuries and neonatal complications such as bronchopulmonary dysplasia and IVH lead to long-term outcomes like cognitive difficulties, problems with executive function, and memory issues. Neuroplasticity, to some extent, may help mitigate the neurological outcomes, but children face educational challenges like ADHD and ASD. Additional socioeconomic factors, such as maternal education and access to quality healthcare, also influence these developmental outcomes. This affects the social adaptation and overall health in adulthood.

To overcome these challenges, early detection and prevention strategies for developmental issues, along with long-term follow-ups, are crucial. This is evident from the increased survival rate of preterm newborns into adulthood with advances in perinatal care and multidisciplinary management, like promoting safe pregnancies, administering antenatal corticosteroids, and preventing infections during pregnancy. Neonatal care should focus not just on survival but also on the reduction of brain injuries. Early interventions, such as physiotherapy, social skill training, and personalized educational plans, can significantly help preterm children with social and learning difficulties. Parental involvement, such as positive parental support, can improve behavioral outcomes. Ongoing research in neuroimaging, genetics, and precision therapies offers scope for earlier diagnosis and tailoring individualized treatment plans to improve the long-term prognosis for preterm children. However, we need further longitudinal studies to fully understand the cognitive, emotional, and social trends of preterm individuals and ensure that care systems adapt to support their evolving needs into adulthood.
